# The Problem of Heredity

**Published:** 1908-08-22

**Authors:** A. R. Urquhart

**Affiliations:** James Murray's Royal Asylum, Perth.


### Editor's Letter-Box.

### THE PROBLEM OF HEREDITY.

*To the Editor of* The Hospital.

Sir,—It is distinctly encouraging to find a notice of Mr. Heron's paper on the "Statistics of Insanity" in the pages of The Hospital. Those of us who have been urgent to record and investigate the facts of heredity have been fortunate in at least finding competent workers in applied mathematics to bring order out of chaos and to set forth reasoned conclusions which can be appreciated as the results of scientific methods. These results are not matters of personal opinion, subject to bias and the errors of the personal equation—they can be checked and resumed in consideration of further information. Perhaps you might have found space to mention that Mr. Heron's study was based upon the material which has accumulated in my hands during the past thirty years, but that is of little importance further than showing that the conclusions are not derived from a hurried investigation of the questions at issue. Had that been all that I desired to say, I might have let the article in The Hospital pass unnoticed; but, in view of the importance of the subject, I would fain add a few words to invite the attention of the profession to the opportunity which has occurred and the necessities of the case.

Professor Karl Pearson produced his "Grammar of Science" so long ago as 1892. A second edition was called for in 1900. In short, it was recognised as a most important treatise which opened up new ground and afforded new hopes. Quite lately, Mr. Francis Galton founded the Eugenics Laboratory, of which Mr. Heron is Fellow. It is unnecessary to make more than a passing reference to the work which has been done and the material which is rapidly accumulating. The material which is not accumulating in sufficient quantity is the information which the doctors of the country could supply best. It is the record of the family diseases of our country. The facts are hidden away in notebooks and reluctant memories, and the sooner they are rescued from oblivion, arranged and investigated, the sooner we shall arrive at a more scientific understanding of the *corpus* on which we work.

The records of a specialist are not exclusively desirable, the collection of family histories should be gathered from random sources. Dr. John Macpherson has endeavoured to obtain a series of facts on this principle, and, after considerable personal cost, has at length been aided by the Carnegie Trust. The results, which cannot but be valuable, will, no doubt, be given to the scientific world in due course. Yet these encouraging beginnings can only be recognised as tentative. In order to arrive at exact conclusions the facts must be numerous and authentic.

It is hardly credible that the British Medical Association have declined to further this investigation through their Scientific Grants Committee. The importance of the inquiry is eminently justified, the expectation of beneficial results is grounded on experience already attained, and it only remains to awaken interest in such papers as Mr. Heron's in order to emphasise the necessity for further progress while the opportunity remains open.

Our business is to collect data, to weigh and to measure, to draw conclusions, to inform the public, and I might add, in the words of the motto on the title page of the "Grammar of Science," "*La critique est la vie de la Science.*"

It is many years since I concluded that the mere observation that a previous case of insanity had occurred in the family of an insane person is little better than useless for scientific purposes. It is absolutely necessary that all persons should be noted—sane or insane. On the same principle, all families should be recorded—sane or insane, together with the characters of each person, their individual diseases, and so on. I have systematically urged that the records of special hospitals can only be of authentic value provided that the statements are carefully made and that they are relative to the whole circumstance. What can be more similar than the course of rheumatism and the course of insanity? What can be more notorious than the invasion of phthisis among an insane stock? Yet these complete and intimate records, if selected at random, would give information of the deepest interest, not only in reference to rheumatism and phthisis and mental disorders, but would reveal the heredity of all disorders, the rise and fall of families, the facts upon which eugenics must be founded.

I write as a physician, not as a specialist; I urge this great investigation as a citizen, not as a theoriser.

I have shown that the worst possible heredity—the insanity of both parents—is not inevitably productive of damaged offspring. The results of twenty-eight families in this unfortunate category were : 33 per cent. of children alive and sane, 44 per cent. insane, 23 per cent. dead. I have insisted on the regenerative powers of the race, and the beneficial effects of appropriate environment. I trust that a fatalistic acceptation of our present evils will never be the rule for the medical profession. In that hope, therefore, I cry for more light—not the uncertain glimmer of impressions and vague guesses of scattered observers, but rather the effulgence of a triumphant science.

A. R. Urquhart.

James Murray's Royal Asylum, Perth.
August 17, 1908.

